# Supplementary material for: A causal inference approach for estimating effects of non-pharmaceutical interventions during Covid-19 pandemic
Source: PLoS One. 2022 Sep 28;17(9):e0265289. doi: 10.1371/journal.pone.0265289 (PMC9518862; doi:10.1371/journal.pone.0265289)
Supplement: S1 Appendix — (ZIP) [file pone.0265289.s001.zip › S6_Fig.pdf]

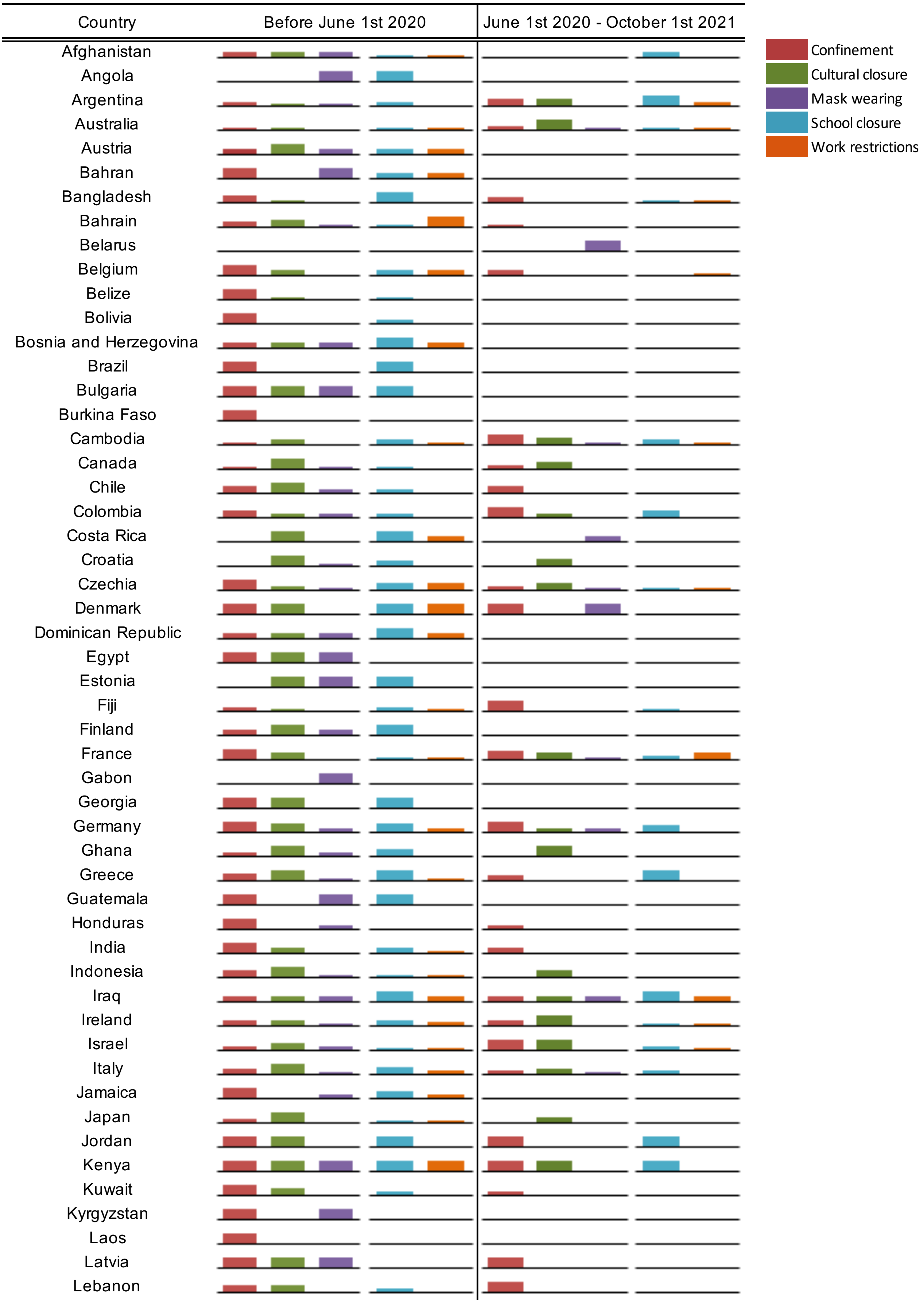
Country
Before June 1st 2020
June 1st 2020 - October 1st 2021
Confinement
Cultural closure
Mask wearing
School closure
Work restrictions
Afghanistan
Angola
Argentina
Australia
Austria
Bahran
Bangladesh
Bahrain
Belarus
Belgium
Belize
Bolivia
Bosnia and Herzegovina
Brazil
Bulgaria
Burkina Faso
Cambodia
Canada
Chile
Colombia
Costa Rica
Croatia
Czechia
Denmark
Dominican Republic
Egypt
Estonia
Fiji
Finland
France
Gabon
Georgia
Germany
Ghana
Greece
Guatemala
Honduras
India
Indonesia
Iraq
Ireland
Israel
Italy
Jamaica
Japan
Jordan
Kenya
Kuwait
Kyrgyzstan
Laos
Latvia
Lebanon

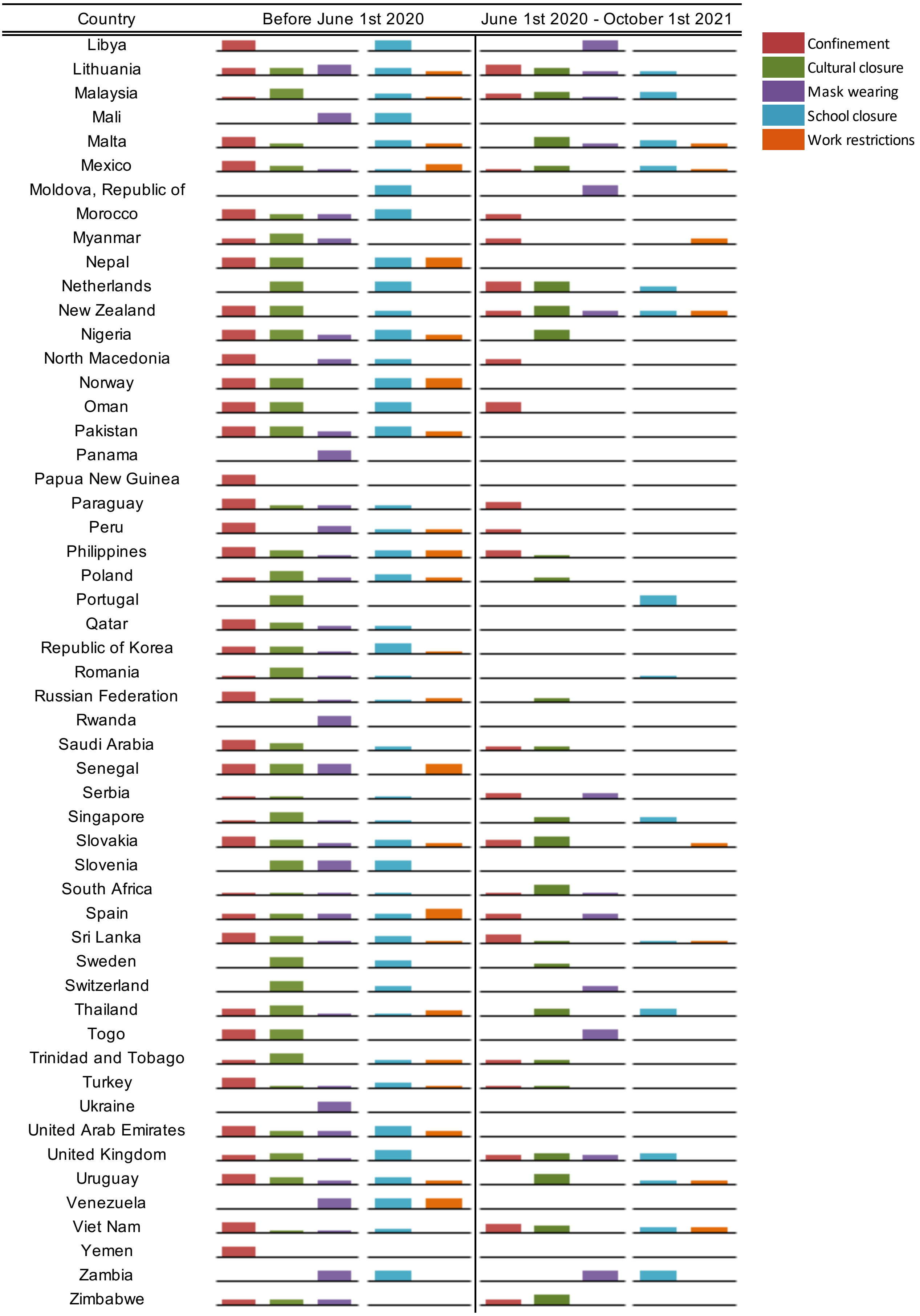
Country
Before June 1st 2020
June 1st 2020 - October 1st 2021
Libya
Lithuania
Malaysia
Mali
Malta
Mexico
Moldova, Republic of
Morocco
Myanmar
Nepal
Netherlands
New Zealand
Nigeria
North Macedonia
Norway
Oman
Pakistan
Panama
Papua New Guinea
Paraguay
Peru
Philippines
Poland
Portugal
Qatar
Republic of Korea
Romania
Russian Federation
Rwanda
Saudi Arabia
Senegal
Serbia
Singapore
Slovakia
Slovenia
South Africa
Spain
Sri Lanka
Sweden
Switzerland
Thailand
Togo
Trinidad and Tobago
Turkey
Ukraine
United Arab Emirates
United Kingdom
Uruguay
Venezuela
Viet Nam
Yemen
Zambia
Zimbabwe
Confinement
Cultural closure
Mask wearing
School closure
Work restrictions

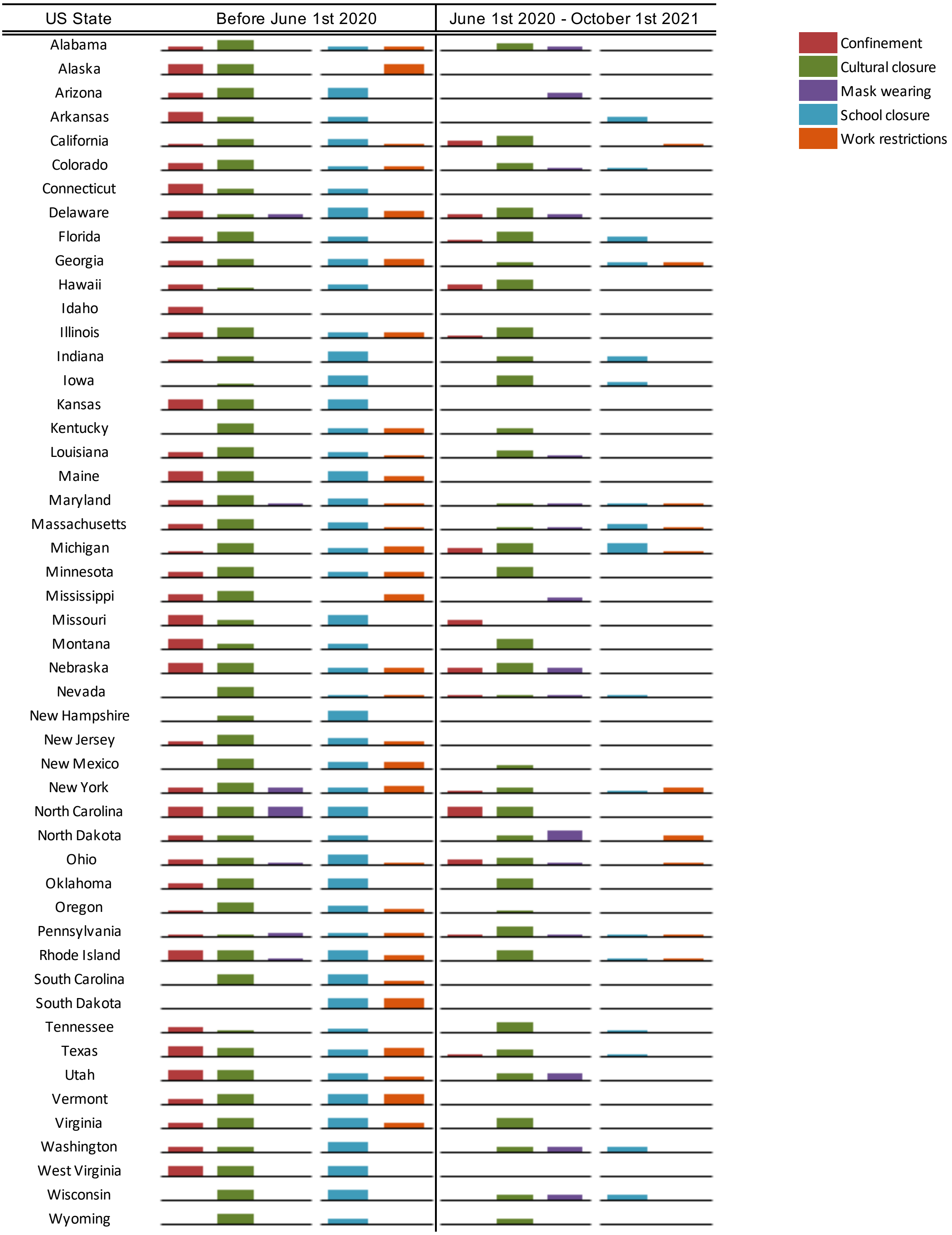
US State
Before June 1st 2020
June 1st 2020 - October 1st 2021
Alabama
Alaska
Arizona
Arkansas
California
Colorado
Connecticut
Delaware
Florida
Georgia
Hawaii
Idaho
Illinois
Indiana
Iowa
Kansas
Kentucky
Louisiana
Maine
Maryland
Massachusetts
Michigan
Minnesota
Mississippi
Missouri
Montana
Nebraska
Nevada
New Hampshire
New Jersey
New Mexico
New York
North Carolina
North Dakota
Ohio
Oklahoma
Oregon
Pennsylvania
Rhode Island
South Carolina
South Dakota
Tennessee
Texas
Utah
Vermont
Virginia
Washington
West Virginia
Wisconsin
Wyoming
Confinement
Cultural closure
Mask wearing
School closure
Work restrictions
