# Supplementary material for: A causal inference approach for estimating effects of non-pharmaceutical interventions during Covid-19 pandemic
Source: PLoS One. 2022 Sep 28;17(9):e0265289. doi: 10.1371/journal.pone.0265289 (PMC9518862; doi:10.1371/journal.pone.0265289)
Supplement: S1 Appendix — (ZIP) [file pone.0265289.s001.zip › Supplemental_material.pdf]

Supporting information

Appendix E1

Clustering of countries based on socio-economic and health variables We characterized each country by a cluster ID representing its socio-economic and health status. We used DBSCAN (Density-Based Spatial Clustering of Applications with Noise) [1] because the number of clusters generated was not limited to a pre-defined constant. The parameters *eps* and *Minpts* were decided based on experimental trials. First, we arbitrarily set five countries to be the minimum number of samples in a neighborhood and used the Euclidian metric to calculate distances between instances. The parameter *eps*, i.e., the maximum distance two countries can be from one another while still belonging to the same cluster, was selected according to a k-dist graph [5]; this graph calculates the distance between a point and its k-th nearest points (in this case, k = 5). We then plotted the distances in ascending order on a k-distance graph and chose the value at the point of maximum curvature, where the graph has the highest slope (1S-A Fig). Finally, noise samples were manually assigned to the defined clusters using domain knowledge. This procedure resulted in four clusters (1S-B Fig). Finally, we one-hot encoded the cluster identifier and represented the socioeconomic status with four binary variables: cluster 1, cluster 2, cluster 3, and cluster 4. The list of features used in the DBSCAN algorithm are detailed in 1S Table with their descriptive statistics.

1S Fig. Clusters performed on socio-economic and health variables. (A) The k-nearest neighbor distances plot, defined as the distance from a point to its k nearest neighbors (here, $k = 5$), was used to pick the parameter *eps* on the point of maximum curvature. Nineteen noisy samples were manually assigned to the defined clusters. (B) Countries are colored by their respective cluster ID. The DBSCAN algorithm found a total of four clusters: a large group comprising mostly African, South/Central American, and South East Asian countries (in dark green); another cluster predominantly with Eastern European countries (light green); the third cluster with the USA, Canada, and part of Western Europe (light orange); and lastly, a group of countries composed of the UK, along with a few Western European and Asian countries (dark orange).

Appendix E2

Robustness of missing values imputation We imputed missing values on the following datasets:

1. Mobility data

   The Google mobility data contains mobility trends within different geographical levels: (i) country region (defined as the entire country), sub-region 1 (regions within the country), and sub-region 2 (districts within the regions). Most of the missing values came from sub-regions 1 and 2, especially in small countries where such fine detailed information was not available. For each country and each mobility category, we computed the daily average mobility across all regions. This corresponds to grouping the data by country and date and taking the mean of a specific mobility category (this operation automatically excludes missing values). The remaining missing values represented a negligible fraction of the dataset

1S Table. Descriptive statistics for observed variables from OWID and the World Bank's WDI datasets (accessed on October 10, 2021) across 113 countries.

Variable	Mean ± std	Observations
**Socio-economic variables**		
Access to electricity	92.36 ± 17.03	% of population
Access to electricity	97.43 ± 6.25	% of urban population
Forest area (%)	30.92 ± 21.25	land in hectares
Air pollution, mean annual exposure	26.91 ± 20.50	micrograms per cubic meter
GDP per capita	23198.36 ± 20778.14	
Human Development Index	0.76 ± 0.13	
People with basic handwashing facilities	59.93 ± 17.64	% of population
Hospital beds	3.32 ± 2.49	per 100,000 people
Population density	234.21 ± 768.68	
Median age	33.05 ± 8.70	
Population ages 65 and above	10.33 ± 6.65	% of total population
Population ages 70 and above	6.60 ± 4.57	% of total population
**Health variables**		
Cardiovascular death rate	242.38 ± 120.20	per 100,000 people
Life expectancy at birth, total	74.59 ± 6.45	in years
Antiretroviral therapy coverage	60.39 ± 14.23	% of people living with HIV
Smoking prevalence, males	32.21 ± 17.64	% of adults
Smoking prevalence, females	12.18 ± 11.39	% of adults
Prevalence of undernourishment	7.33 ± 9.88	% of population
Prevalence of overweight	50.74 ± 6.73	% of adults
Cause of death by communicable diseases and maternal, prenatal and nutrition conditions	16.45 ± 9.58	% of total
Cause of death by non-communicable diseases	74.91 ± 18.31	% of total
Diabetes prevalence	7.8 ± 3.97	% of population ages 20 to 79
Mortality from CVD, cancer, diabetes or CRD	17.72 ± 5.82	% of ages 30 to 70

(from 1% to a maximum 3% of the entire data), corresponding to a few mobility categories that were not recorded on a certain day in specific countries. Therefore, we imputed such missing values with a linear interpolation, which is a robust imputation method for time series data.

2. Socio-economical and health variables

   In this dataset, we imputed missing values with the mean of all samples. From a total of 23 socio-economical and health variables considered in our study, 6 of them had missing information in some countries, according to 2S Table.

2S Table. Percentages of missing values in socio-economical and health covariates.

Feature name	Percentage of countries with missing values (out of 113 countries)
People with basic handwashing facilities	61.06 (69/113)
Smoking prevalence in males	14.2 (16/113)
Smoking prevalence in females	14.2 (16/113)
Hospital beds per 100,000 people	4.4 (5/113)
Population ages 70 and above	0.88 (1/113)
Prevalence of undernourishment (% of population)	9.73 (11/113)

We conducted a robustness analysis on the mean imputation of the missing features by performing imputation using the k-Nearest Neighbors (KNN) instead. We picked the number of neighboring samples as the default value (n_neighbors = 5). After KNN, we performed DBSCAN clustering (using the same approach as described in Appendix E1) and compared the final clusters. We found that for both imputation methods, the final clusters encountered by DBSCAN resulted in the same set of clusters. Additionally, we checked the correlation between the 23 socio-economical covariates. We observed that in general, features were collinear. For instance, the feature with the highest percentage of missing values, "People with basic handwashing facilities", had a Pearson correlation coefficient higher than 0.7 with features like "Access to electricity" ($p < 0.001$), "Human development index" ($p < 0.001$) and "Cause of death by NCD" ($p < 0.001$). Thus, imputing missing values in a small subset of the features should not affect the outcome predictions or the final causal effect estimates.

2S Fig. Analysis of socio-economical and health features' missing values imputation using a K-nearest neighbors (KNN) model. (A) Resulting DBSCAN clusters after imputing missing values with KNN. For both imputation methods (average and KNN), the final clusters encountered by DBSCAN resulted in the same set of countries (1S-B Fig). (B) Heatmap correlation matrix across features. Features generally showed collinearity between each other, suggesting that imputation should not affect the final results.

Appendix E3

Geography selection The geography was selected based on the intersection of countries with available NPI data, mobility trends and at least 70% non-missing variables from socioeconomical and health databases (World Development Indicators

[WDI] and Our World in Data [OWID]). The final dataset consisted of the following 113 countries:

Afghanistan, Angola, United Arab Emirates, Argentina, Antigua and Barbuda, Australia, Austria, Belgium, Burkina Faso, Bangladesh, Bulgaria, Bahrain, Bahamas, Bosnia and Herzegovina, Belarus, Belize, Bolivia, Brazil, Barbados, Canada, Switzerland, Chile, Colombia, Cabo Verde, Costa Rica, Czechia, Germany, Denmark, Dominican Republic, Egypt, Spain, Estonia, Finland, Fiji, France, Gabon, United Kingdom, Georgia, Ghana, Greece, Guatemala, Honduras, Croatia, Hungary, Indonesia, India, Ireland, Iraq, Israel, Italy, Jamaica, Jordan, Japan, Kenya, Kyrgyzstan, Cambodia, Republic of Korea, Kuwait, Laos Peoples Democratic Republic, Lebanon, Libya, Sri Lanka, Lithuania, Luxembourg, Latvia, Morocco, Moldova, Republic of, Mexico, North Macedonia, Mali, Malta, Myanmar, Mongolia, Mauritius, Malaysia, Nigeria, Netherlands, Norway, Nepal, New Zealand, Oman, Pakistan, Panama, Peru, Philippines, Papua New Guinea, Poland, Portugal, Paraguay, Qatar, Romania, Russian Federation, Rwanda, Saudi Arabia, Senegal, Singapore, Serbia, Slovakia, Slovenia, Sweden, Togo, Thailand, Trinidad and Tobago, Turkey, Ukraine, Uruguay, United States, Venezuela, Viet Nam, Yemen, South Africa, Zambia, Zimbabwe.

Because we had more locally fine-grained mobility and NPI data available for the United States, we used the state-level information of all 50 states.

Appendix E4

Outcome prediction by Gradient Boosting Machines During the first experiments of our study, we considered three possible regression algorithms for outcome prediction: (i) support vector machines (SVM), (ii) ridge regression, and (iii) XGBoost, a Python implementation of gradient boosting machines. None of the first two models outperformed XGBoost. Gradient boosting machines do not try to fit a linear model, they support regularization, and are designed to handle missing data with built-in features. However, they are known for such disadvantages as overfitting and a lack of interpretability. To make the models more interpretable, we analyzed feature contributions using the Tree SHAP algorithm. To avoid overfitting, we tuned the XGBoost's hyperparameters with a 5-fold cross-validation on the training dataset via a randomized grid search. Since our models generated slightly different performances solely based on the random seed used to initialize the optimization procedure, we trained 100 models in different subsamples of the training set (bootstrap samples) for each time lag, and we reported the MSE with the 95% confidence interval. Parameters of the best performance models using grid search can be found in the table below, where we show one example of the runs for each outcome and time lag.

3S Table. Parameters of the best performance models using grid search. For all XGBoost models, we used a grid search approach to find the optimal `min_child_weight` [1, 2, 3, 5, 7], `gamma` [0, 0.1, 0.2, 0.3, 0.4], `colsample_by_tree` [0.3, 0.4, 0.5, 0.7, 1.0], `learning_rate` [0.10, 0.20, 0.30, 0.40, 0.50] and `max_depth` [3, 5, 7, 9]. All models were trained and tested with 70% and 30% of the dataset, respectively. The best model was chosen based on the configuration that gave the lowest MSE on the validation set.

Outcome	Time lag	`min_child_weight`	`gamma`	`colsample_by_tree`	`learning_rate`	`max_depth`
Residential mobility	7	2	0.3	1.0	0.2	9
	14	5	0.4	0.4	0.3	7
	21	7	0.4	0.7	0.1	9
R_t	7	3	0.4	0.5	0.1	7
	14	2	0	0.4	0.5	9
	21	1	0.1	0.5	0.2	9

Appendix E5

Identification assumptions The Rubin Causal Model [2] used in this study is an approach to causal inference that is based on a framework of potential outcomes. Under this framework, causal effects are estimated with comparisons of potential outcomes under the two different treatments: one that received the intervention, and another that received a different intervention (e.g., placebo or no treatment). In our context, these two groups are defined as days in countries where NPIs were imposed vs. days in countries where NPIs were not imposed. Causal estimates from this source are unbiased when three assumptions are met, namely (i) conditional exchangeability, (ii) Stable Unit Treatment Value Assumption (SUTVA) and (iii) positivity.

Conditional exchangeability states that the counterfactual outcomes are conditionally independent of the treatment given the set of covariates. Under this assumption, the treatment groups are "exchangeable", i.e., there are no unmeasured confounders that are a common cause of both treatment and the outcome [6]. In other words, if all confounders are measured, then we can assume that exchangeability holds within the strata dictated by the confounders, and we can estimate the causal effect by using methods that eliminate the confounding (e.g.,, by emulating a randomized controlled trial [RCT] using balancing weights methods). This is because randomization ensures that the covariates associated with the outcome are equally distributed between the treatment groups. In reality, in observational studies as the one we studied here, one cannot empirically verify that conditional exchangeability holds or there is no unmeasured confounding. Thus, causal inference relies on subject-matter knowledge to identify possible confounders in the data, so that the assumption is at least approximately true.

SUTVA stipulates that the outcome of one unit should not be affected by another unit's treatment assignment. Although SUTVA plays a central role in the identification of causal effects, this assumption does not hold in many settings. For example, when a certain country bans inbound flights from its neighbouring countries (introduction of a new NPI), the observable outcome (mobility change, $\mathrm{R_t}$ rate or any other Covid-19 morbidity measure) of the banned countries are certainly affected by this treatment assignment. Another classical example is given in epidemiology, where the possibility of an individual becoming infected depends on whether the population is vaccinated. In our study of NPI effectiveness, SUTVA is an unrealistic assumption. Yet, we claim that our final estimated effects are still a good enough approximation. We direct the reader to the work of Hudgens and Halloran [7]. In their paper they do a review of previous studies that estimated causal effects in the presence of interventions' interference.

Positivity, also called a lack of covariate overlap, is the assumption that any sample has a positive probability of receiving all values of the treatment variable. In other words, to identify causal effects pertaining to a treatment A, there must be some probability of receiving A given a certain baseline of covariates X, otherwise treatment versus control causal effects cannot be identified. Mathematically, $Pr(A = a|X = x) > 0$ for all x where $Pr(X = x) \neq 0$. The positivity assumption has the very important consequence of ensuring that features in both treatment groups are equal in their distribution. In our work, we empirically verify to some extent whether positivity holds by checking whether the distribution of covariates is similar between the two treatment groups. By inspecting the success of the weighting method used (in our case, either IPW or the AdvBal algorithm [3], we are ensuring that positivity holds in our dataset. In the next section, we discuss the balancing evaluation of the applied weights in more detail.

Appendix E6

Balancing evaluation A fundamental step required to produce reliable effect estimates is to control for systematic differences between the treatment and control groups. To this end, balancing weights methods, such as adversarial balancing (AdvBal) [3], generate weights that reduce the observed confounding biases, and thus can be used to emulate a randomized controlled trial (RCT) by re-weighting the population. To evaluate the performance of AdvBal, we analysed the distribution of all covariates across the treatment groups before and after re-weighting samples with AdvBal. The covariate balancing plot in 3S Fig is an example of an evaluation plot when work restrictions was assigned as treatment. In the plot, the difference in distribution between treatment groups is quantified by the absolute standard mean difference (ASMD), defined as the absolute value in the difference in means of a covariate between the treatment groups, divided by its standard deviation in the treated group. A small ASMD value represents a good balance, while a value larger than some threshold is considered imbalanced. In this study, we considered a threshold of 0.1 as a reasonable cutoff for acceptable ASMD [4]. We observed that before balancing, many features were biased between the treated and untreated groups. AdvBal was able to minimize this discrepancy, bringing the ASMD to less than 0.1 for all covariates.

3S Fig. Balancing evaluation plots of AdvBal algorithm (upper) and IPW (lower) of the dataset during train phase. The plot displays the absolute standard mean difference (ASMD) of each feature in the original unweighted data (orange triangles) and in the weighted data (blue circles) obtained with the weights generation method. Although both methods were able to balance all covariate distributions and bring ASMD below 0.1, AdvBal performed marginally better.

Appendix E7

NPIs effect on places of recreation The mobility data for retail and recreation areas represents the change (relative to the period before the pandemic) in the number of visitors to places like restaurants, shopping centers, and libraries. The estimated causal effects show that, apart from mask wearing, all social distancing policies were effective in decreasing visits to this category of places (4S Fig). Of these, school and cultural mandates were the most effective ones, achieving a 50.4% [95% CI: 41.9%, 58.2%] and 47.4% [95% CI: 39.4%, 55.1%] average reduction in 21 days, respectively. Overall, changes in mobility outside residential areas were larger in magnitude.

4S Fig. Effect of NPIs over time on retail and recreation areas. Model results for the 113 countries show that school and cultural mandates were the most effective ones, achieving a 50.4% and 47.4% reduction in the average number of visitors to recreation areas in 21 days, respectively. AdvBal: Adversarial balancing algorithm, IPW: inverse propensity weighting.

Appendix E8

Complementary analysis of effect of NPIs In an alternative cohort study design, the treatment group is composed of all events of a certain NPI of interest, whereas the control group contains all events of the remaining NPIs. Thus, instead of estimating the effect of NPIs compared to a period when no NPIs are imposed, we aimed at estimating the effects of individual NPIs relative to others. We consider this scenario a more

"strict" design where we hoped to examine whether our findings would be consistent with the results of the original study.

Results of this complementary analysis are shown in 5S Fig. We found that compared to other NPIs, school closure was the most effective restriction in changing the two mobility categories 14 days later after its initiation. It was also the only NPI that greatly impacted R_t in the same time period.

5S Fig. Estimated causal effects of NPIs under the study design "NPI vs. other NPIs". Under this approach, we investigated which NPI has the highest impact compared to the others. AdvBal: Adversarial balancing algorithm, IPW: inverse propensity weighting.

Appendix E9

6S Fig. NPI employment statistics per country and US state before and after June 1st 2020. Until June 1^{1st}, governments worldwide enacted restrictions in a more consistent way, i.e., the distribution of the NPIs employed was more similar across countries.

References

1. Ester M, Kriegel HP, Sander J, Xu X, et al. A density-based algorithm for discovering clusters in large spatial databases with noise. In: kdd. vol. 96; 1996. p. 226–231.
2. Rubin DB. Estimating causal effects of treatments in randomized and nonrandomized studies. Journal of educational Psychology. 1974;66(5):688.
3. Ozery-Flato M, Thodoroff P, Ninio M, Rosen-Zvi M, El-Hay T. Adversarial balancing for causal inference. arXiv preprint arXiv:181007406. 2018;.
4. Stuart EA, Lee BK, Leacy FP. Prognostic score–based balance measures can be a useful diagnostic for propensity score methods in comparative effectiveness research. Journal of clinical epidemiology. 2013;66(8):S84–S90.
5. Gaonkar MN, Sawant K. AutoEpsDBSCAN: DBSCAN with Eps automatic for large dataset. International Journal on Advanced Computer Theory and Engineering. 2013;2(2):11–16.
6. Hernán MA, Robins JM. Causal inference; 2010.
7. Hudgens MG, Halloran ME. Toward causal inference with interference. Journal of the American Statistical Association. 2008;103(482):832–842.
