# Supplementary figures and images for: A causal inference approach for estimating effects of non-pharmaceutical interventions during Covid-19 pandemic
Source: PLoS One. 2022 Sep 28;17(9):e0265289. doi: 10.1371/journal.pone.0265289 (PMC9518862; doi:10.1371/journal.pone.0265289)

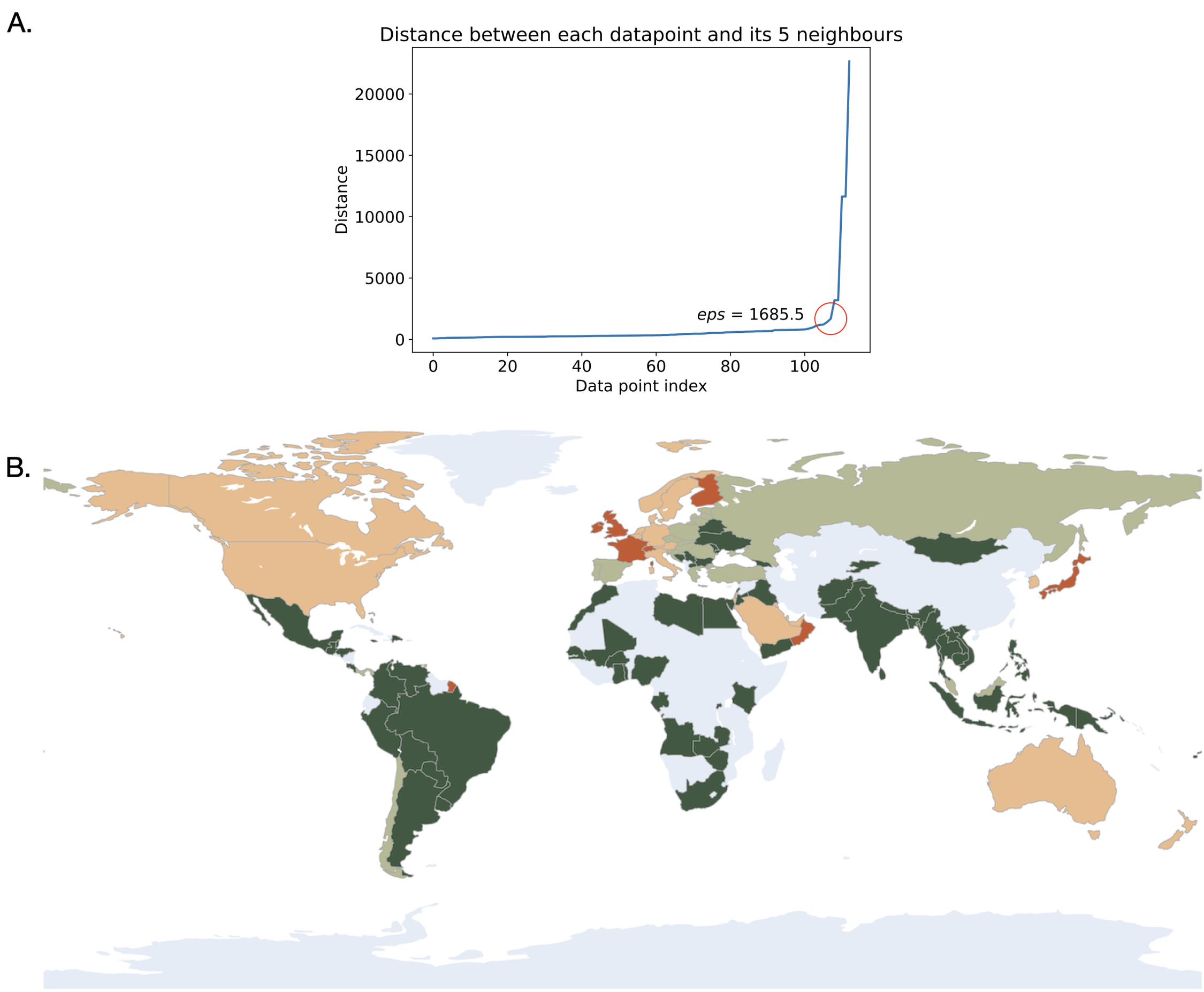

Supplement: S1 Appendix — (ZIP) [file pone.0265289.s001.zip › S1_Fig.tiff]

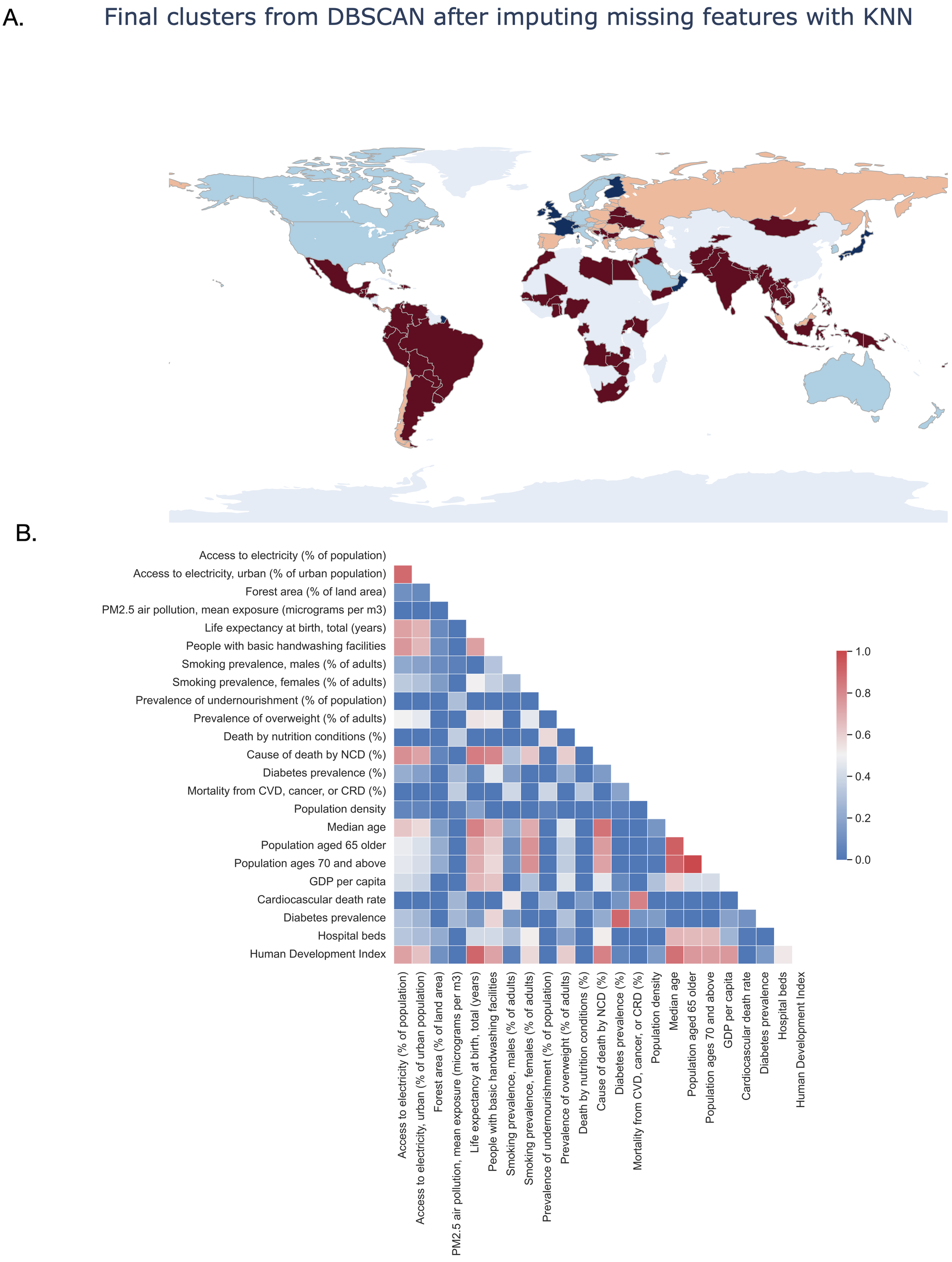

Supplement: S1 Appendix — (ZIP) [file pone.0265289.s001.zip › S2_Fig.tiff]

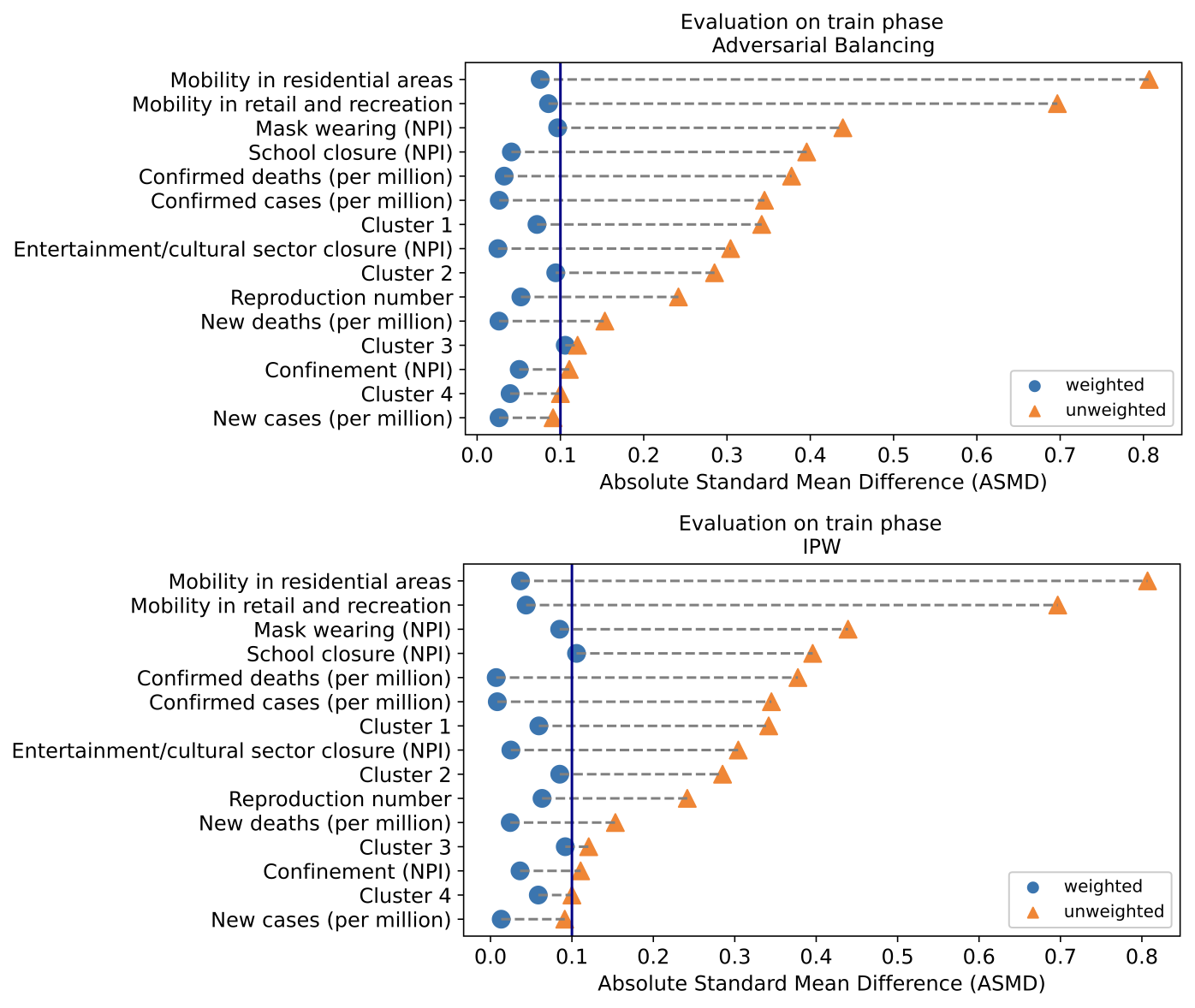

Supplement: S1 Appendix — (ZIP) [file pone.0265289.s001.zip › S3_Fig.tiff]

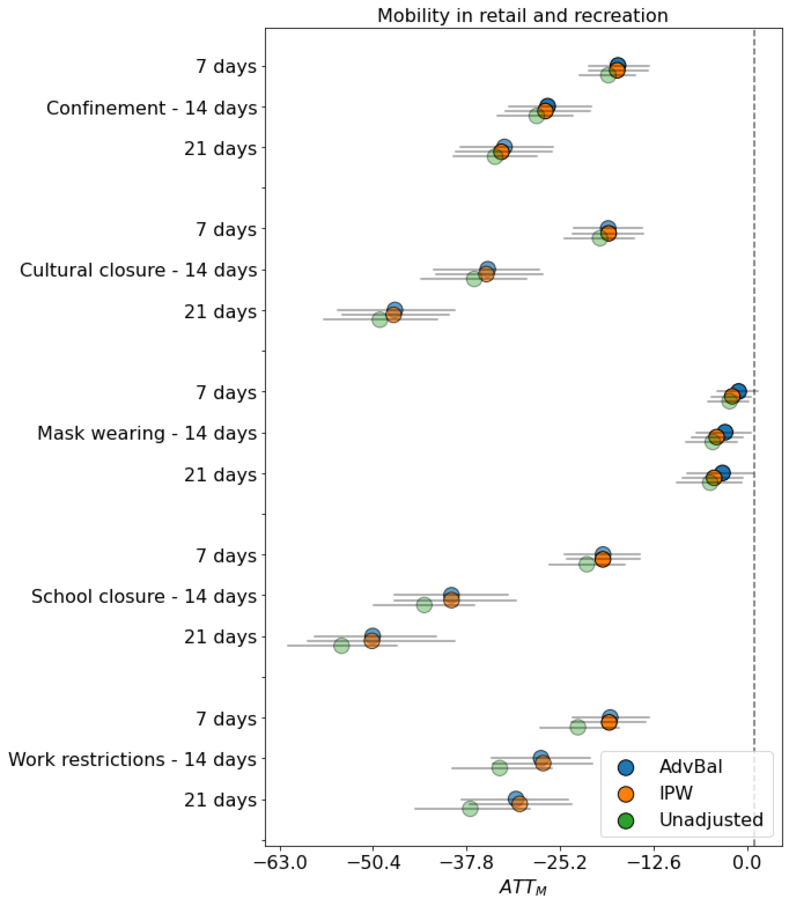

Supplement: S1 Appendix — (ZIP) [file pone.0265289.s001.zip › S4_Fig.tiff]

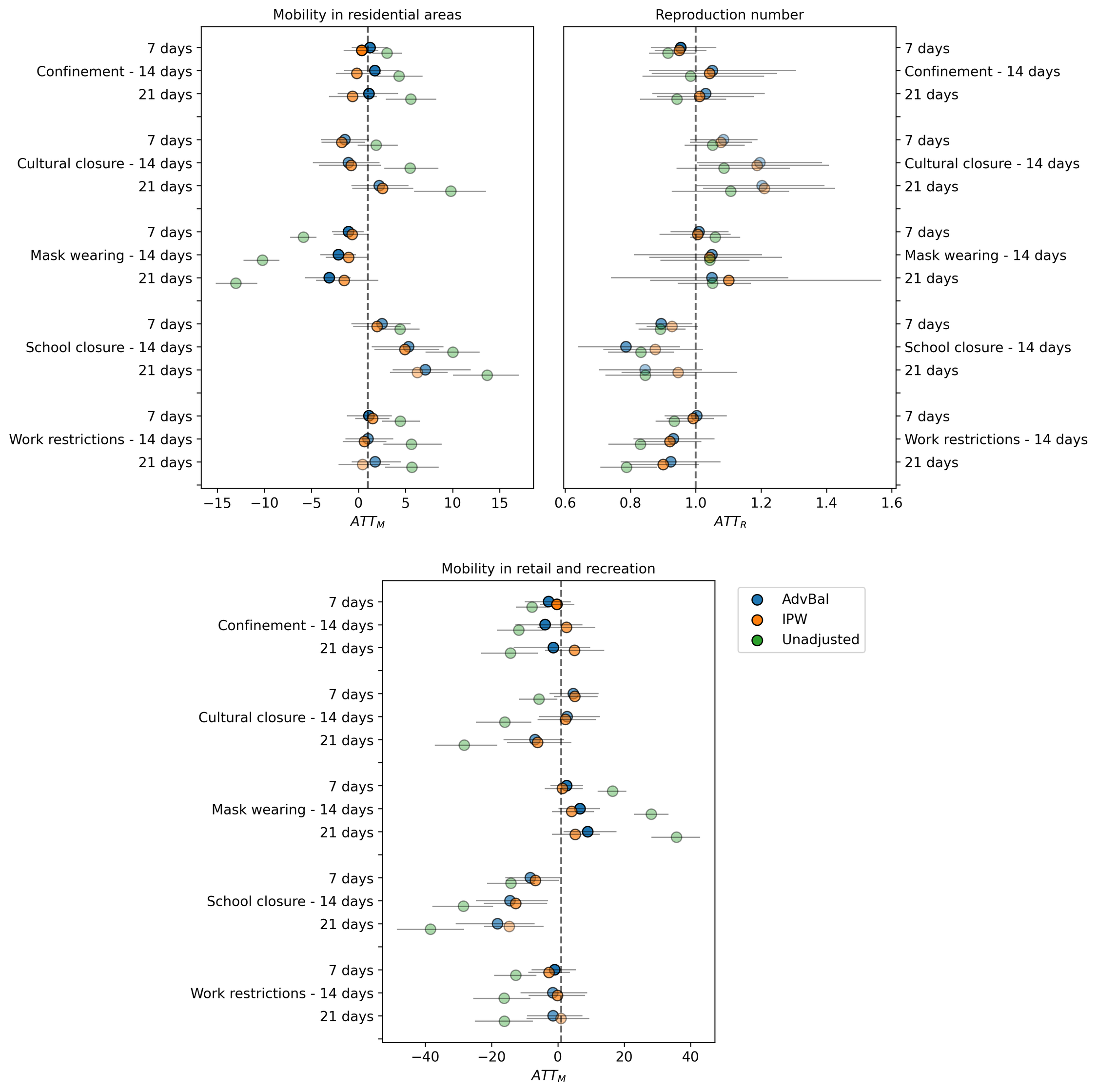

Supplement: S1 Appendix — (ZIP) [file pone.0265289.s001.zip › S5_Fig.tiff]
